# October 2026—academization of psychotherapy training in Austria

**DOI:** 10.3389/frhs.2026.1829383

**Published:** 2026-09-23

**Authors:** Sara Plimon-Rohm, Thomas Probst

**Affiliations:** 1 Federal Ministry for Labour, Social Affairs, Health, Care and Consumer Protection, Vienna, Austria; 2 Department of Psychology, University of Salzburg, Salzburg, Austria; 3 Faculty of Psychology, University of Vienna, Vienna, Austria

**Keywords:** academization, Austria, psychotherapy, training, universities

## Abstract

According to the Psychotherapy Act 2024, a new psychotherapy training program will start in Austria in October 2026. This is a paradigm shift towards the academization of psychotherapy training. The new psychotherapy training program consists of three phases: various qualifications as the first training phase, a new master's program in psychotherapy at universities as the second training phase (500 study places at public universities across Austria will be funded annually by the government), and a postgraduate third training phase in one of the accredited psychotherapeutic methods or clusters at a licensed psychotherapeutic professional association. The new psychotherapy training program could reduce financial costs for trainees compared to the old psychotherapy training system in Austria. Moreover, the new master's program in psychotherapy may lead to more professorships and academic staff for psychotherapy at universities and university outpatient psychotherapy clinics. This could boost psychotherapy research in Austria with international visibility. Despite these opportunities, there remain challenges to be addressed in the future. This perspective describes the new psychotherapy training program in Austria and discusses its opportunities and problems.

## 1 Introduction

In Austria, the first Psychotherapy Act 1990 regulated the two phases of psychotherapy training consisting of general training, the “Propädeutikum,” followed by specialized training, the “Fachspezifikum.” The “Propädeutikum” comprises at least 765 h of theory and at least 550 h of practical work. The trainees then select one of the licensed psychotherapy methods (see below) for the “Fachspezifikum,” where trainees have to complete at least 300 h of theory and at least 1,600 h of practical work. In Austria, the two phases “Propädeutikum” and “Fachspezifikum” have to be completed at licensed psychotherapeutic professional associations, and the trainee has to cover all the financial costs of this training regime, as has been the case over the last three decades.

The new Psychotherapy Act 2024 [PThG 2024 (1)] introduced a new academized psychotherapy training program, consisting of the following three phases.

- Various qualifications are considered the first training phase. For more details, see Section 1.1.
- The second training phase is a new master's program in psychotherapy with 120 European Credit Transfer and Accumulation System (ECTS) points (one ECTS point corresponds to 25 h of total student workload in Austria). For more details, see Section 1.2.
- The third phase is postgraduate training specific to a licensed psychotherapeutic professional association in one of the 23 accredited psychotherapy methods in Austria or in one of the four psychotherapeutic clusters into which the 23 methods can be categorized (2) (psychoanalytic–psychodynamic cluster: analytical psychology, group psychoanalysis, individual psychology, psychoanalysis psychoanalytically oriented psychotherapy, autogenic psychotherapy, daseinsanalysis, dynamic group psychotherapy, hypnotherapy, catathym-imaginative psychotherapy, concentrative movement therapy, transactional analysis; humanistic cluster: existential analysis, existential analysis and logotherapy, Gestalt-theoretical psychotherapy, integrative Gestalt therapy, integrative therapy, client-centered psychotherapy, person-centered psychotherapy, psychodrama; systemic cluster: neuro-linguistic psychotherapy, systemic family therapy; behavioral therapy method/cluster). In Austria, the psychotherapeutic professional associations have been licensed in recent decades to provide training in psychotherapeutic methods. Therefore, the third phase takes place within one of the 23 accredited methods until psychotherapeutic professional associations are licensed for a psychotherapeutic cluster in the future (except for behavioral therapy, which is already both a method and a cluster). For more details, see Section 1.3.
- At the end of the third phase, the trainees have to complete a psychotherapeutic state exam (“Approbationsprüfung”) organized by the Austrian federal ministry responsible for healthcare.

When offered at public universities, the first two phases incur no financial costs for students (except for tuition fees at the universities and the costs of completing the standardized test to apply for a study place in the new master's program). The third phase, however, incurs financial costs for the trainees as it is not financed by the public. This division of training can also be found in the medical specialties and in the training to become a clinical psychologist or healthcare psychologist in Austria. Only after completion of all three phases and the psychotherapeutic state exam is one eligible to apply to be listed on the professional list of psychotherapists and then allowed to treat patients with psychotherapy independently.

The “new” psychotherapy training according to PThG 2024 begins on 1 October 2026. During the transition period, the former psychotherapy training system according to the first Psychotherapy Act from 1990 is regulated in §§ 60 and 61 PThG 2024 as follows: the “Propädeutikum” has to be completed by 30 September 2030, and the “Fachspezifikum” can be started no later than 1 October 2030 and must be finished by 30 September 2038.

The aims of this Perspective article are to provide a summary of all relevant sources in one document, to illustrate the new distribution of responsibilities for psychotherapy training in Austria, and to discuss the opportunities and tensions generated by the academization of the profession.

### 1.1 First phase of the new psychotherapy training program

The first phase of the new psychotherapy training program is a bachelor's degree according to §§ 10 and 11 PThG 2024, including the Appendix to the PThG 2024 (3), or qualifications defined as equivalent in § 10 (2) PThG 2024 (see Table 1). This is in line with the traditional heterogeneity of options to enter psychotherapy training in Austria.

**Table 1:** Characteristics of the first psychotherapy training phase.

Type of degree	Specifications
Bachelor's degree in psychotherapy according to § 11 PThG 2024 and Appendix	General basis training in psychotherapy, introduction to the four clusters, core subjects of psychotherapy, interdisciplinary subjects, research methods, scientific work, professional and methodological understandings, an understanding of professional ethics, basic scientific skills, basic social communicative and self-reflection skills through theoretical training, practical psychosocial experiences, psychotherapeutic supervision, and psychotherapeutic self-experience
§ 10 (2) 1 PThG 2024 Diploma or bachelor's and master's degree in medicine	
§ 10 (2) 2 PThG 2024 Bachelor's degree in psychology	
§ 10 (2) 3 PThG 2024 Bachelor's degree in social work	
§ 10 (2) 4 PThG 2024 Master's degree in social work	The curricula include 60 ECTS points of a bachelor's study program in social work
§ 10 (2) 5 PThG 2024 Master's degree in social education/social pedagogy	The curricula include 60 ECTS points of a bachelor's study program in social work
§ 10 (2) 6 PThG 2024 Co-responsible practice of music therapy	§ 8 and § 19 Music Therapy Act, MuthG, Federal Law Gazette I nr. 93/2008
§ 10 (2) 7 PThG 2024 Authorization to practice a higher-level medical-therapeutical-diagnostical profession	§ 1 Federal law on the higher-level medical-therapeutical-diagnostical professions, MTD-Act 2024, Federal Law Gazette I nr. 100/2024
§ 10 (2) 8 PThG 2024 Authorization to practice a higher-level health and nursing care profession	§ 27 Health and Nursing Care Act, Federal Law Gazette I nr. 108/1997
§ 10 (2) 9 PThG 2024 Authorization to practice the midwifery profession	§ 10 Midwifery Act, Federal Law Gazette nr. 310/1994
§ 10 (2) 10 PThG 2024 Bachelor's degree in counselling	Regulation on Life and Social Counselling, Federal Law Gazette II nr. 116/2022

### 1.2 Second phase of the new psychotherapy training program

The second phase of the new psychotherapy training is a master's program in psychotherapy with 120 ECTS points. Five hundred study places per year are financed by the government at seven locations/ten public universities:

- 140 in Graz (University of Graz and Medical University of Graz)
- 100 in Innsbruck (University of Innsbruck and Medical University of Innsbruck)
- 80 in Krems on the Danube (University for Continuing Education Krems)
- 50 in Linz (University of Linz)
- 50 in Salzburg (University of Salzburg)
- 40 in Vienna (University of Vienna and Medical University of Vienna)
- 40 in Klagenfurt (University of Klagenfurt)

It is widely assumed that there will be more applications than there are study places available in the new master's program. Therefore, a standardized admission procedure is applied at the 10 participating public universities. Interested individuals have to complete an online self-assessment (information about the study program and the profession of psychotherapy) and apply to their preferred university. Then a standardized paper-and-pencil test must be completed on a set day (the same 3-h test on the same day across Austria). This test will include basic knowledge of psychotherapy, psychology, verbal-analytical thinking, socio-emotional competencies, and the ability to understand English (4). At each location, the applicants with the best performance in the standardized test will be considered for a study place in the master's program in psychotherapy. However, a quota system will be applied: at least 75% of the study places are reserved for individuals with an Austrian high school diploma (or equivalent) and at least 95% of the study places are for EU citizens (5). The main source of information is a website of the participating public universities (6).

The participating universities have agreed to align their curricula for the master's program in psychotherapy with the framework curriculum developed by the Austrian Psychological Society (7) in cooperation with the public medical universities and departments/faculties of psychology at the public universities (see Table 2). The framework curriculum for the master's program in psychotherapy is based on § 12 PThG 2024 and the Appendix to the PThG 2024. This framework curriculum focuses on combining theoretical and practical aspects of psychotherapy training and on integrating the four clusters of psychotherapy. Integrating all four clusters of psychotherapy allows students to gain knowledge about all four clusters, and this is necessary for the psychotherapeutic state exam (“Approbationsprüfung”) as well as for making an informed decision about which cluster/method to pursue during the third training phase. It is unknown whether private universities and universities of applied sciences, which are also able to offer the second phase, will adhere to this framework curriculum or develop their own curricula according to § 12 PThG 2024 and the Appendix to the PThG 2024.

**Table 2:** Framework curriculum for the master's program in psychotherapy developed by the Austrian Psychological Society.

Module/block	Content
Psychopathology and psychotherapeutic methods	Development of psychological structures, processes, and functions Psychopathology, psychiatry, and psychosomatics (including somato-psychic interrelations) Introduction to the four psychotherapeutic clusters Special psychotherapeutic settings (e.g., therapy for children, group therapy, online therapy, forensic contexts, current and future cross-cluster and cluster-specific developments)
Diagnostics	Fundamentals of diagnostics and expert report preparation Diagnostic classification systems Cluster-specific psychotherapeutic diagnostics in theory and practice
Psychotherapy research	Methods of psychotherapy research Findings from international psychotherapy research regarding the psychotherapeutic clusters Research internship (cluster-specific)
Regulatory frameworks and non-psychotherapeutic practice	Legal and ethical frameworks of psychotherapy Psychopharmacology/psychopharmacotherapy and other non-psychotherapeutic methods (e.g., clinical psychological treatment, physical activity interventions)
Practical exercises in psychotherapeutic methods	Cross-cluster practical exercises (e.g., crisis intervention, modular-based psychotherapy) Cluster-specific practical exercises
Psychotherapeutic practical components	Internship [at least 10 ECTS points in psychiatry (inpatient or day-clinic settings)] Psychotherapeutic supervision (cluster-specific) Psychotherapeutic self-experience (cluster-specific)
Master's thesis	Master's thesis supervision seminar Master's thesis

### 1.3 Third phase of the new psychotherapy training program

The third phase is postgraduate training in a psychotherapeutic method or cluster at a licensed psychotherapeutic professional association regulated by §§ 13–19 PThG 2024 and the “Psychotherapy Training, State Exam, and Quality Assurance Regulation” (PTh-AAQV 2024) (8). The psychotherapeutic state exam is held at the end of the third phase (§ 18 PThG 2024). According to § 5 (2) of the PTh-AAQV 2024, the third phase of training must include at least the following:

- 1,000 h of psychotherapeutic treatment (at least 500 h of psychotherapeutic work with moderately and/or severely ill patients) with at least 200 h of psychotherapeutic supervision;
- 400 h of theory;
- 200 h of psychotherapeutic self-experience;
- 150 h of preparation for and completion of the psychotherapeutic state exam; and
- 100 h of individual specialization.

According to § 17 (1) PThG 2024, psychotherapeutic treatment under supervision may begin only after the trainee has completed 5,000 h, equivalent to 200 ECTS points, of relevant workload. The recommended qualification profile comprises 90 ECTS points of psychotherapy foundations and basic psychotherapeutic experience, 80 ECTS points of coherent cluster-/method-specific specialization, and 30 ECTS points for the master's thesis, defense, and any additional specialization (9). According to this recommendation, the total 120 ECTS points from the second training phase, i.e., the master's program in psychotherapy, will be credited towards these 200 ECTS points if the second training phase has been completed at public universities in Austria (9). Future documents regarding the third phase of the new psychotherapy training program will be published on the website of the Austrian federal ministry responsible for healthcare (in the psychotherapists section) as the central information source (10). It is also important to note that the third training phase can be entered not only after completing a master's program in psychotherapy (second phase of the new psychotherapy training) but also with other qualifications listed in § 10 (3) PThG 2024 (for example, psychologists who completed their postgraduate training in clinical psychology in Austria or psychiatrists).

## 2 Sources and scope

As the documentary basis of this article, the following legislation, regulations, framework curricula, official government documents, and institutional sources were consulted:

- The Psychotherapy Act 2024 (1). https://www.ris.bka.gv.at/Dokumente/BgblAuth/BGBLA_2024_I_49/BGBLA_2024_I_49.html
- Appendix to the Psychotherapy Act 2024 (3). https://www.ris.bka.gv.at/Dokumente/BgblAuth/BGBLA_2024_I_49/Anlagen_0001_478E0AD1_44AF_4B1B_B4D1_E1E35E15E8C6.html
- A website of the participating universities regarding the admission procedure for the new second training phase (6). https://psychotherapiestudieren.at/
- Framework curriculum of the Austrian Psychological Society for the new second training phase (7). https://www.oegps.at/_files/ugd/437743_29f87035a29e43489f8cea8222140fb1.pdf
- Regulatory document for the new third training phase named “Psychotherapy Training, State Exam, and Quality Assurance Regulation” (PTh-AAQV 2024) (8). https://www.ris.bka.gv.at/Dokumente/BgblAuth/BGBLA_2024_II_292/BGBLA_2024_II_292.html
- Recommendation for the new third training phase regarding the training content that is necessary before trainees can treat patients under supervision “Empfehlung zu § 17 Abs. 1 PThG 2024” (9). https://www.sozialministerium.gv.at/dam/jcr:a2fba623-79a8-4da3-8cdf-3f8d5aa2dc8a/Empfehlung%20%C2%A7%2017%20Abs.%201%20PThG%202024%20(Stand%20Mai%202026).pdf

The period covered is from 30 April 2024 (the date of the new Psychotherapy Act) to 23 August 2026 (the date of finalizing this Perspective article). The date on which these online sources were last consulted was 23 August 2026.

These sources are considered authoritative and sufficient to describe the reform of psychotherapy training in Austria, as they are documents published by the government, the participating universities, and ministries. This article combines an analysis of official sources with the informed professional knowledge of the authors regarding the reform.

The first author, SP-R, works within the responsible unit of the Federal Ministry for Labour, Social Affairs, Health, Care and Consumer Protection responsible for the Psychotherapy Act, especially the third training phase. As current vice president of the Austrian Psychological Society, the last author, TP, is involved in coordinating the psychotherapy program across public universities.

Some of these documents (e.g., admission procedure, curriculum) may change after experience with the first admission procedures and/or the first master's programs in psychotherapy has led to changes being considered necessary. Moreover, the new second phase is currently financed until the end of 2027 and depending on future financing, universities may change their current curricula.

## 3 Discussion

The PThG 2024 brings about a paradigm shift in psychotherapy training in Austria. This new psychotherapy training starting in October 2026 will include three phases. If trainees complete the first and second phases at public universities, they will incur no financial costs for these phases (apart from tuition and Students' Union fees). One aim of the “new” psychotherapy training is to make the profession more socially accessible, especially for individuals with fewer financial resources, as the “old” psychotherapy training (Propädeutikum, Fachspezifikum) had to be privately financed. Yet, even in the new psychotherapy training program, the third phase must be financially covered by the trainees, as no public financing exists.

A central aim of the Psychotherapy Act 2024 is the academization of psychotherapy in Austria (11, 12). Broader implications of the academization concern the relationship between scientific training and clinical competence, between standardization and therapeutic pluralism, and between professional autonomy and quality assurance. This academization has the potential to establish a research training environment to realize the scientist-practitioner model in Austria, a model considered to be ideal (13, 14). Scientist-practitioners can be scientifically active in their psychotherapeutic practice, integrate new scientific findings into psychotherapeutic practice, and make their own practical work accessible to science (13, 14).

In this context, the new master's program in psychotherapy can be seen as a milestone, since psychotherapy is the last health profession in Austria to be academized. Due to the new master's program in psychotherapy, there may be more professorships for psychotherapy at universities. This offers many opportunities to strengthen the international visibility of psychotherapy research in Austria, especially when university outpatient psychotherapy clinics are established to offer optimal conditions for psychotherapy training and research. The use of the same data collection procedures across university outpatient clinics may offer large datasets on psychotherapy processes and outcomes, as in other countries (15).

Yet, it should be kept in mind that these outcomes are plausible but not guaranteed by the reform. One needs to clearly distinguish between legally mandated outcomes and future expectations that still depend on decisions made by universities, professional associations, or insurers. Future indicators to assess the reform's impact could be the number of academic positions, funded projects, publications, clinical trials, multicenter networks, and patient outcomes. Only some of the unresolved challenges in the new psychotherapy training system can be mentioned here. First, patients treated by trainees working under supervision receive no reimbursement from social health insurance at present. This may make it difficult for the trainees to reach the required 1,000 h of psychotherapeutic treatment under supervision in the third training phase, compared with the 600 h required in the old training system, as patients must be found who are willing to cover all the financial costs of their psychotherapeutic treatment themselves (trainees usually have to offer treatment at lower rates per session to attract enough patients). However, this rise in hours is explained by the higher overall quality of the new program. Second, the third phase of the new psychotherapy training program is still financially costly for the trainees, and it would be welcome to have public funding for the third phase, especially for trainees from a lower socioeconomic background. Third, more than 500 study places will probably be necessary in the future, as the number of psychotherapists finishing psychotherapy training in Austria has risen to over 500 in the last few years, and, even more importantly, the increase in mental health problems requires enough professionals to offer timely care. These places could be offered by private universities, universities of applied sciences, and fee-based courses at public universities (university extension courses). Fourth, employment options with salaries for trainees in clinics during the third training phase are scarce.

Currently, the most important open questions from the perspective of students are as follows: How expensive will the third training phase be after the master's program? How likely is it that students will be able to obtain one of the 500 study places for the master's program (depending on the number of applications at the universities)?

From the perspective of the professional associations, important open questions comprise the following: How can both systems be provided in parallel, maintaining the old system (Propädeutikum, Fachspezifikum) within the transition period and, at the same time, establishing the new third phase of training? Will the role of professional associations be transformed?

To summarize, the new psychotherapy training in Austria offers great opportunities, but challenges remain to be addressed and potential unintended consequences such as excessive homogenization of therapeutic methods or conflicts between cross-disciplinary integration and methodological specialization need to be monitored.

## Data Availability

The original contributions presented in the study are included in the article/Supplementary Material, further inquiries can be directed to the corresponding author.

## References

[1] Psychotherapiegesetz 2024 (PThG 2024). BGBl. I Nr. 49/2024 (2024) Available online at: https://www.ris.bka.gv.at/Dokumente/BgblAuth/BGBLA_2024_I_49/BGBLA_2024_I_49.html (Accessed August 23, 2026)

[2] Bundesministerium für Soziales, Gesundheit, Pflege und Konsumentenschutz. Patientinnen- und Patienten-Information über die psychotherapiewissenschaftlichen Ausrichtungen (2024). Available online at: https://www.sozialministerium.gv.at/dam/jcr:067ed3c8-aaea-4c84-84c2-a3afb9cef836/Patienteninformation_(BMGSPK),_Stand_29.04.2020.pdf (Accessed August 23, 2026)

[3] Anlage zum Psychotherapiegesetz 2024 (PThG 2024). BGBl. I Nr. 49/2024 (2024). Available online at: https://www.ris.bka.gv.at/Dokumente/BgblAuth/BGBLA_2024_I_49/Anlagen_0001_478E0AD1_44AF_4B1B_B4D1_E1E35E15E8C6.html (Accessed August 23, 2026)

[4] Available online at: https://psychotherapiestudieren.at/Testvorbereitung/ (Accessed August 23, 2026)

[5] Available online at: https://psychotherapiestudieren.at/Kontingentregelung/ (Accessed August 23, 2026)

[6] Available online at: https://psychotherapiestudieren.at (Accessed August 23, 2026)

[7] Available online at: https://www.oegps.at/_files/ugd/437743_29f87035a29e43489f8cea8222140fb1.pdf (Accessed August 23, 2026)

[8] Psychotherapie-Ausbildungs-, Approbationsprüfungs- und Qualitätssicherungs-Verordnung 2024 (PTh-AAQV 2024). 292. Verordnung des Bundesministers für Soziales, Gesundheit, Pflege und Konsumentenschutz über die Psychotherapie-Ausbildung, Approbationsprüfung und Qualitätssicherung (2024). Available online at: https://www.ris.bka.gv.at/Dokumente/BgblAuth/BGBLA_2024_II_292/BGBLA_2024_II_292.html (Accessed August 23, 2026)

[9] Bundesministerium für Arbeit, Soziales, Gesundheit, Pflege und Konsumentenschutz. Empfehlung zu § 17 Abs 1 PThG 2024 (2026). Available online at: https://www.sozialministerium.gv.at/dam/jcr:a2fba623-79a8-4da3-8cdf-3f8d5aa2dc8a/Empfehlung%20%C2%A7%2017%20Abs.%201%20PThG%202024%20(Stand%20Mai%202026).pdf (Accessed August 23, 2026)

[10] Berufe A bis Z, Berufslisten und Gesundheitsberuferegister – Berufe P: Psychotherapeutin, Psychotherapeut. Available online at: https://www.sozialministerium.gv.at/Themen/Gesundheit/Medizin-und-Gesundheitsberufe/Berufe-A-bis-Z,-Berufslisten-und-Gesundheitsberuferegister.html (Accessed August 23, 2026)

[11] Datler W Drossos A Gornik E Korunka C. Akademisierung der Psychotherapie: Aktuelle Entwicklungen, Historische Annäherungen und Internationale Perspektiven. Vienna: Facultas (2023).

[12] Hochgerner M Plimon-Rohm S. Die gesetzliche Neuregelung der Psychotherapie in Österreich. Feedback. (2024) 2:51–71. 10.30820/2752-2245-2024-2-51

[13] Schmelzer D. Scientist-Practitioner-Modell. In: Stumm G Pritz A, editors. Wörterbuch der Psychotherapie. Vienna: Springer (2000) p. 614–615. 10.1007/978-3-211-99131-2_1671

[14] Gelso CJ. On the making of a scientist-practitioner: a theory of research training in professional psychology. Train Educ Prof Psychol. (2006) 5(1):3–16. 10.1037/1931-3918.S.1.3

[15] Velten J Christiansen H Hoyer J In-Albon T Lincoln T Lutz W. Effectiveness of cognitive behavioral therapy for adult mental disorders: a large-scale naturalistic study across 29 university outpatient clinics. Behav Res Ther. (2025) 186:104691. 10.1016/j.brat.2025.104691 39919361

